# Effects of thoracic nerve block on perioperative lung injury, immune function, and recovery after thoracic surgery

**DOI:** 10.1002/ctm2.38

**Published:** 2020-07-08

**Authors:** Wei Zhang, Xuhui Cong, Liyuan Zhang, Mingyang Sun, Bing Li, Hongfang Geng, Jianqin Gu, Jiaqiang Zhang

**Affiliations:** ^1^ Department of Anesthesiology and Perioperative Medicine Center for Clinical Single Cell Biomedicine Henan Provincial People's Hospital People's Hospital of Zhengzhou University Zhengzhou Henan P. R. China; ^2^ Department of General Medicine Henan Provincial People's Hospital People's Hospital of Zhengzhou University Zhengzhou Henan P. R. China

**Keywords:** dexmedetomidine, immune, lung injury, paravertebral, recovery, thoracic surgery

## Abstract

**Background:**

To investigate the effects of thoracic nerve block on perioperative lung injury, immune function, and recovery after thoracic surgery

**Methods:**

A total of 120 patients with lung cancer were randomly allocated into three groups: general anesthesia group (GAL group), thoracic paravertebral nerve block (TPVB) combined with general anesthesia (TPL group), and TPVB (with paravertebral dexmedetomidine) combined with general anesthesia group (TDL group); 120 patients with esophageal cancer were randomly allocated into three groups: general anesthesia group (GAE group), TPVB combined with general anesthesia group (TPE group), and thoracic epidural block combined with general anesthesia group (TEE group). Lung injury and immune function were evaluated. Hemodynamic changes, early recovery in post‐anesthesia care unit, pain, 6‐min walking test (6MWT), drug consumption, and life quality were also observed. The duration in the PACU of patients was retrospectively analyzed. The effect of dexmedetomidine on lung injury was established in vitro.

**Results:**

The lung injury, including injury scores, apoptosis, and inflammation, were decreased in the TDL group compared with the GAL group and TPL group. The ratio of CD4^+^/CD8^+^ cells at the end of surgery was higher in the TPE group than in the GAE group. More stable hemodynamic was found in TPL group and TPE group. Acute pain was alleviated and the 6MWT was enhanced by TPVB with or without dexmedetomidine. Anesthetic consumption was decreased by thoracic nerve block.

**Conclusions:**

Thoracic nerve block, especially TPVB with or without paravertebral dexmedetomidine, can enhance recovery after thoracic surgery. Protection against independent lung injury and cellular immune dysfunction may be a potential mechanism.

## Graphical abstract

Thoracic paravertebral nerve block with or without dexmedetomidine protects against the lung injury and immune dysfunction during pneumonectomy and esophagectomy.

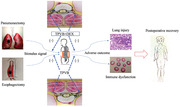

**Abbreviations**
ASA: American Society of Anesthesiologists
DEX: dexmedetomidine
HIF‐1α: hypoxia inducible factor‐1α
LIRI: lung ischemia reperfusion injury
OLV: one‐lung ventilation
TEA: thoracic epidural anesthesia
TPVB: thoracic paravertebral block

## 1 BACKGROUND

Thoracic surgery is widely performed worldwide. Lung cancer and esophageal cancer have a high incidence and account for a large proportion of thoracic surgeries. Although surgery is an important treatment, it has the characteristics of a high incidence of pain, severe stress response, increased inflammation, decreased immune function, and high incidence of pulmonary complications.^[^ ^1^, ^2^, ^3^ ^]^ Postoperative recovery after surgery is hampered by these factors. Though multiple causative factors are involved in the mechanism, the improvement of anesthesia quality is playing an increasingly important role.^[^ ^4^ ^]^

Effective nerve blockade could be produced by thoracic paravertebral nerve block (TPVB) without causing severe hemodynamic changes.^[^ ^5^ ^]^ It is helpful to reduce perioperative pain^[^ ^6^ ^]^ and adverse outcomes.^[^ ^7^ ^]^ Dexmedetomidine (DEX) has been proven to inhibit stress levels and inflammation in a one‐lung ventilation model.^[^ ^8^ ^]^ It is unclear whether the combination of TPVB and DEX could reduce lung injury and improve immune function.

 This study intended to evaluate the application of TPVB combined with DEX on recovery after thoracic surgery. Tissue samples and blood samples were collected to determine lung injury, inflammation, and cellular immune function by using flow cytometry, Western blotting, TUNEL staining, ELISA, and other technical methods. A retrospective study was conducted to assess the effect of TPVB on the duration in the PACU. To further determine the effect of dexmedetomidine on lung injury, an in vitro ischemia‐reperfusion injury model was established. In addition, a retrospective analysis of the duration in the PACU was conducted. Our study proves that recovery after thoracic surgery could be enhanced by TPVB with or without dexmedetomidine. The protection against mitochondrial injury in independent lung injury and cellular immune dysfunction may be a potential mechanism.

## 2 MATERIALS AND METHODS

### 2.1 Trial design

A randomized, double‐blind study was designed to enroll 320 patients (including lung cancer and esophageal cancer patients) from October 2019 to February 2020.

### 2.2 Participants

A total of 160 patients with lung cancer and 160 patients with esophageal cancer, aged 18 to 65 years, ASA I to II, BMI < 30 kg/m^2^, were selected.

Inclusion criteria: confirmed preoperative diagnosis; no history of diabetes, blood disease, and other metabolic disorders; no history of hormone use; no autoimmune disease; no chronic obstructive or (and) restrictive lung disease; FVC > 80% of the expected value; and FEV1 > 70% of the expected value.

Exclusion criteria: preoperative lung infection; disorders in communication; inability to cooperate with researchers; history of preoperative chemoradiotherapy; severe cardiovascular and cerebrovascular disease; previous history of other surgery; refusal to participate in the trial; severe hypoxemia during surgery (SpO_2_ remaining below 90% for 1 min after F_i_O_2_ is adjusted to 100%); data loss; intraoperative blood transfusion; and surgical procedure conversion.

### 2.3 Study settings

The study was approved by the Ethics Committee of Henan Provincial People's Hospital (2019‐41). Data were collected from Henan Provincial People's Hospital.

### 2.4 Interventions

After written informed consent was obtained, patients with lung cancer were randomly allocated into three groups through a digital table: general anesthesia group (GAL group), thoracic paravertebral nerve block combined with general anesthesia (TPL group), and thoracic nerve block (mixed with dexmedetomidine) combined with general anesthesia group (TDL group).

After written informed consent was obtained, patients with esophageal cancer were randomly allocated into three groups through a digital table: general anesthesia group (GAE group), thoracic paravertebral nerve block combined with general anesthesia group (TPE group), and thoracic epidural block combined with general anesthesia group (TEE group).

### 2.5 Preoperative preparations and anesthesia protocol

Blinding principle was well controlled in the present study. First, patients were delivered into the pre‐anesthesia room. An anesthesiologist who was blinded to this study prepared the drugs and TPVB or TEE. All the catheter insertion sites were covered with a film dressing to conceal which approach was being used.

After the patient entered the room, ECG and SpO_2_ were routinely monitored; the peripheral venous access of the upper limbs was opened, the radial artery was punctured and placed under local anesthesia, and arterial blood pressure was monitored. The BIS was monitored using an EEG dual spectrum index monitor.

#### 2.5.1 Thoracic paravertebral block

TPVB was administered before anesthesia induction. Following the localized infiltration of 1% lidocaine, the parathoracic long‐axis in‐plane technique for puncture was performed. According to the location of the surgical incision, the two‐point method (T_4‐5_ and T_6‐7_) was selected, and 10 mL of anesthetic was injected at each point. Ten minutes after the injection was completed, the anesthesia effect was tested to determine whether the nerve block was successful.

The local anesthetic in the TDL group was 0.5 µg/kg DEX mixed with ropivacaine (the final concentration of ropivacaine was 0.5%); the local anesthetic in the TPL and TPE groups was 0.5% ropivacaine.

Thoracic epidural anesthesia was performed before the induction of anesthesia. The epidural puncture was placed between T_6‐7_. Three milliliters of 2% lidocaine was administered to the epidural space for a test injection. Ten milliliters of 0.375% ropivacaine (AstraZeneca, Wilmington, DE, USA) was injected after the effect of epidural anesthesia was determined.

#### 2.5.2 Anesthesia induction

After the intravenous injection of 0.08‐0.12 mg/kg midazolam, 0.2‐0.6 mg/kg etomidate, 0.1‐1.0 µg/kg sufentanil, and 0.6‐0.9 mg/kg rocuronium, a double‐lumen bronchial tube was inserted, and fiber bronchoscopy was used to confirm the position. The mechanical ventilation parameters in one‐lung ventilation (OLV) were as follows: FiO_2_ 70‐100%, oxygen flow 1.0‐1.5 L/min, VT 6‐8 mL/kg, RR 10‐14 breaths/min, PEEP 0‐5 cm H_2_O, P_ET_CO_2_ 35‐45 mm Hg, and P_peak_ < 25 cm H_2_O.

#### 2.5.3 Anesthesia maintenance

All patients were intravenously infused with propofol, remifentanil, and *cis*‐atracurium to maintain anesthesia; the propofol infusion rate was adjusted to maintain BIS 40‐50, and HR and MAP fluctuations did not exceed 20% of the baseline value. The intravenous infusion of sodium lactate Ringer's solution was 3‐5 mL/kg/h, and patient‐controlled intravenous analgesia pumps were connected to patients for postoperative analgesia. A total of 1 µg/kg of oxycodone was used in our study for postoperative pain control.

### 2.6 Measurements

#### 2.6.1 Lung cancer patients

A postoperative follow‐up was performed by an anesthesia nurse who was blinded to this study protocol and collected the following information: acute pain at rest and during movement at postoperative day 1 (POD‐1), postoperative day 2 (POD‐2), and postoperative day 3 (POD‐3); chronic pain at 2 months after surgery; assessment of patient quality of life using the EuroQoL 5 Dimensions 5 Questionnaire (EQ score); and use of propofol and remifentanil perioperatively. A 6‐min walking test (6MWT) was conducted by a respiratory therapist who was blinded to this study group on the day before surgery (Pre), POD‐1, POD‐2, and POD‐3. The result of 6MWT was recorded.

#### 2.6.2 Lung specimen acquisition and detection

Lung specimen at least 5 cm away from the edge of the tumor was harvested as quickly as possible after the tumor tissue was excised. Tumor infiltration of the lung tissue specimen was excluded by an independent pathologist. According to different testing purposes, the samples were treated with liquid nitrogen or 4% formaldehyde.

#### 2.6.3 Injury scores in the lung

The injury of the lung specimen was evaluated through light microscopy by a pathologist who was blinded to the study. Lung injury was quantified using a 4‐point scoring system.^[^ ^9^ ^]^

#### 2.6.4 Apoptosis by TUNEL assay

According to the manufacturer's protocol (Roche, Basel, Switzerland), the apoptosis was detected by using TUNEL assay method. The ratio of number of apoptotic cells and total number of cells was defined as the apoptotic index (AI; %).

#### 2.6.5 Inflammation in the lung specimen

The supernatant was obtained after the lung tissues homogenates was produced and centrifuged. According to the manufacturer's instructions, the concentrations of TNF‐α and IL‐6 were measured.

#### 2.6.6 Expression of proteins in the lung specimen

According to the manufacturer's instructions, expressions of relevant proteins were measured by western blot. Lung tissue were incubated with rabbit polyclonal antibodies against hypoxia‐inducible factor‐1α (HIF‐1α), mitochondrial outer membrane 20 (Tom20), iron‐sulfur cluster assembly enzyme‐2 (ISCU2), Bcl‐2, Beclin‐1, LC3II, and BNIP3 (Santa Cruz Biotechnology, Inc., Dallas, TX, USA).

#### 2.6.7 Esophageal cancer patients

The visual analog scale (VAS) and Riker Sedation‐Agitation Scale (SAS) were used to assess patients at the following time points: 20 min after extubation, 6 h postoperatively, POD‐1 and POD‐2. The use of remifentanil and propofol was recorded.

### 2.7 Blood sample acquisition and detection

Venous blood samples were obtained at the following time points: 30 min before anesthesia induction, the end of the surgical procedure, POD‐1 and POD‐2. A three‐channel flow cytometer (FC500; Beckman Coulter, Hialeah, America) was used to analyze the survival of T cell subsets (cluster of differentiation CD3^+^, CD4^+^, CD8^+^, and CD4^+^/CD8^+^).

Concentrations of cortisol (Cor), interleukin‐6 (IL‐6), IL‐4, tumor necrosis factor‐β (TNF‐β), and interferon‐γ (IFN‐γ) were measured with ELISA (R&D Systems, Minneapolis, MN, USA).

### 2.8 Outcomes

The primary outcome was the independent lung injury score in the lung cancer surgery and the ratio of CD4^+^/CD8^+^ at postoperative 24 h (POD‐1) in the esophageal cancer surgery.

The second outcome in the lung cancer surgery was: the apoptosis, inflammation, and expression of proteins in the lung tissue; acute and chronic postoperative pain scores; postoperative patient quality of life; the intro‐operative anesthetic consumption; post‐operative walking distances by 6‐min walking test; the MAP and heart rate (HR) at the following time points: pre (immediately before anesthesia induction), T_1_ (60 min after the surgery start), T_2_ (the end of the surgery), T3(5 min after extubation in PACU); the use of vasoactive drugs, recovery time, and extubation time were recorded.

The second outcome in the esophageal cancer surgery was: percentage of CD3^+^, CD4^+^, and CD8^+^ T cell subsets; the postoperative visual analog scale (VAS) and Riker Sedation‐Agitation Scale (SAS); the intro‐operative anesthetic consumption; the MAP; and heart rate were recorded at the time points consistent with lung cancer surgery. The use of vasoactive drugs, recovery time, and extubation time were recorded.

### 2.9 Sample size calculation

#### 2.9.1 Sample size of patients with lung cancer

The sample size was calculated using an online software “Power and Sample Size.com.” Based on our preliminary study, the scores of independent lung injury during one lung ventilation was 7.0 ± 1.7. Assuming a 20% difference with an alpha‐error of 0.05 (the alpha‐error was corrected by three pairwise comparisons) and a power of 0.8, the minimum number was 31 patients in each group was needed. Allowing for dropouts, we aimed to recruit 40 patients in each group with a total of 120 patients.

#### 2.9.2 Sample size of patients with esophageal cancer

The sample size was calculated using an online software “Power and Sample Size.com.” Based on our preliminary study, the CD4^+^/CD8^+^ at POD‐1 was 0.58. Assuming a 20% difference with an alpha‐error of 0.05 (the alpha‐error was corrected by three pairwise comparisons) and a power of 0.8, the minimum number was 34 patients in each group was needed. Allowing for dropouts, we aimed to recruit 40 patients in each group with a total of 120 patients.

### 2.10 Retrospective data

The clinical data of patients who underwent radical lung cancer and esophageal cancer surgery from October 2016 to October 2019 were retrospectively reviewed. The duration in the PACU was set as an endpoint, and the cohort was established based on the application of TPVB or not. ASA grade, age, gender, education, smoking history, hypertension, diabetes, immune system disease, operation time, blood loss volume, previous nonthoracic surgery history, intraoperative blood transfusion, intraoperative vascular activity drug application, and minimally invasive surgery were regarded as exposure factors.

### 2.11 In vitro cell experiment

#### 2.11.1 Cell preparation

Human alveolar type II epithelial cells (A549) were added to freshly prepared F12K culture medium (sugar) containing 10% fetal bovine serum, 100 U/mL penicillin, and 100 µg/mL streptomycin. A549 cells were routinely cultured in a 37°C and 5% CO_2_ normoxic incubator.

#### 2.11.2 Model establishment

A549 cells were divided into three groups using a random number table method: normoxic culture group (control group), hypoxia/reoxygenation group (LIRI group), and the hypoxia/reoxygenation+dexmedetomidine group (DEX group). The model was established as follows: the medium of A549 cells in logarithmic growth phase was replaced with serum‐free F12K medium and incubated in a normoxic incubator for 1 h. Then, the medium was replaced with a sugar‐free OGD solution and the cells were placed in a hypoxic incubator for 6 h. After that, the OGD solution was replaced with serum‐free F12K medium again, and the cells were reoxygenated for 24 h in a normoxic incubator. In the DEX group, dexmedetomidine was added at the beginning of model establishment at a final concentration of 1 nmol/L.

### 2.12 In vitro measurements

#### 2.12.1 Morphology

After the cell model was established, the growth status and morphological changes of A549 cells were observed by microscopy. After being washed with PBS, centrifuged, and fixed with 3% glutaraldehyde, A549 cells were evaluated by transmission electron microscopy.

#### 2.12.2 Apoptosis

After the cell model was established, pulmonary epithelial cells in the logarithmic growth phase were collected and seeded at a density of 2 × 10^5^ cells/well in a six‐well plate. After the cells adhered completely, they were processed for 48 h. Apoptosis was measured according to the manufacturer's protocol (Biyuntian Annexin V‐FITC Apoptosis Detection Kit).

#### 2.12.3 Reactive oxygen species

After the cell model was established, the reactive oxygen species assay kit fluorescent probe DCFH‐DA was used to detect reactive oxygen species (ROS).

#### 2.12.4 Mitochondrial membrane potential

Cultured pulmonary epithelial cells were resuspended in medium at 1 × 10^6^/mL; rhodamine 123 staining solution was added at 5 µg/mL; and flow cytometry was used to detect the fluorescence intensity at 488 and 525 nm.

### 2.13 Statistical analysis

The statistical analysis was performed using SPSS software version 21.0 (IBM Corp., Armonk, NY, USA). Depending on data distribution, the continuous variables are recorded as mean ± SD or median (interquartile range (IQR)). Two independent sample *t*‐tests were used to compare the continuous variables with normal distribution. To compare non‐normal continuous variables, Kruskal‐Wallis test was used. Frequency and percentage were recorded as categorical variables, which was analyzed using Chi‐square test or Fisher exact test. The median of the duration in the PACU, which was expressed as binary data, was set as the end event. The possible risk factors were analyzed using univariate analysis. The risk factors with *P*‐value < .1 were collected and then analyzed with multivariate regression method.

## 3 RESULTS

### 3.1 Clinical trial

A total of 120 patients with lung cancer were recruited and were randomized into three groups. Seven patients, including three patients in the GAL group, two patients in the TPL group, and two patients in the TDL group, were excluded. A total of 120 patients with esophageal cancer were initially recruited and were randomized into three groups. Ten patients, including four patients in the GAE group, four patients in the TPE group, and two patients in the TEE group, were excluded. After follow‐up, data from the three groups were collected for statistical analysis. No differences were found between the two groups in demographic and operative data, as shown in Table 1.

**TABLE 1:** Characteristics of the patients

	Lung cancer				Esophageal‐cancer			
	GAL Group	TPL Group	TDL Group	*P*‐value	GAE Group	TPE Group	TEE Group	*P*‐value
Age(y)	50 ± 13	56 ± 15	55 ± 10	.174	60 ± 4.2	58 ± 6.35	57.4 ± 7	.174
Gender				.528				.512
Male	19	18	21		25	27	23	
Female	22	24	20		17	13	20	
BMI (kg/m^2^)	23.74 ± 3.16	24.03 ± 2.19	22.14 ± 7.03	.17	23.35 ± 2.6	23.16 ± 1.6	22.02 ± 1.8	.271
ASA classification				.922				.25
ASA I	5	7	6		26	21	28	
ASA II	36	35	35		16	19	15	
Operative duration (min)	153 ± 16	148 ± 27	149 ± 32	.83	205 ± 37	184 ± 31	172 ± 38	.31
Anesthesia duration (min)	199 ± 24	188 ± 27	192 ± 40	.58	210 ± 42	200 ± 36	190 ± 455	.38
OLV_specimen resection_	115 ± 13	111 ± 24	116 ± 28	.71				
Urinary output (ml)	172 ± 18	172 ± 24	179 ± 36	.62	736 ± 127	756.30 ± 235.7	687.32 ± 154.4	.34
Fluid administration	649 ± 84	615 ± 90	596 ± 46	.10	699 ± 168	690 ± 167	634 ± 134	.17
Vasoactive drugs	10/37	3/38	4/38	.04	12/36	4/36	11/38	.03
Recovery time (min)	20 ± 8	19 ± 5	18 ± 6	.62	21 ± 7	19 ± 5	19 ± 5	.49
Extubation time (min)	27 ± 7	23 ± 5	24 ± 7	.27	31 ± 6	30 ± 6	28 ± 6	.23

BMI, body mass index; ASA, American Society of Anesthesiologists; OLV, one lung ventilation; OLV_specimen resection_, the duration from OLV starting to the specimen resection.

### 3.2 Lung cancer patients

#### 3.2.1 Pain relief after lung surgery

At POD‐1, the VAS at rest and movement was decreased in the TPL and TDL groups compared with the GAL group. The VAS of movement at POD‐2 was lower in the TPL and TDL groups than in the GAL group (Figure 2A).

FIGURE 1.1Study flow diagram for lung cancer

**Figure:**
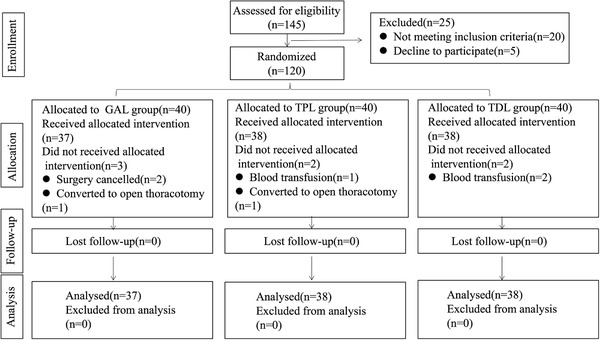

**Figure:**
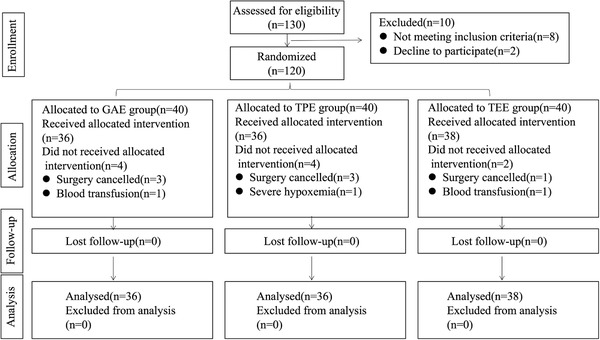

**FIGURE 1.2:**
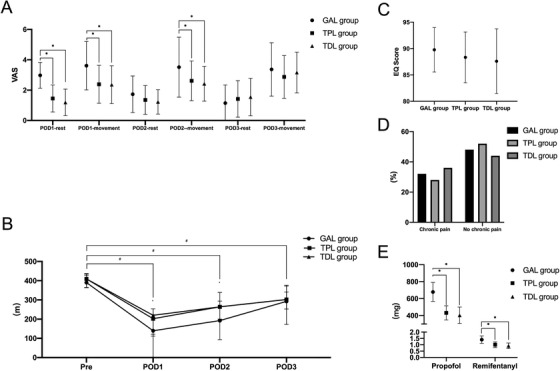
Study flow diagram for esophageal cancer

**FIGURE 2:**
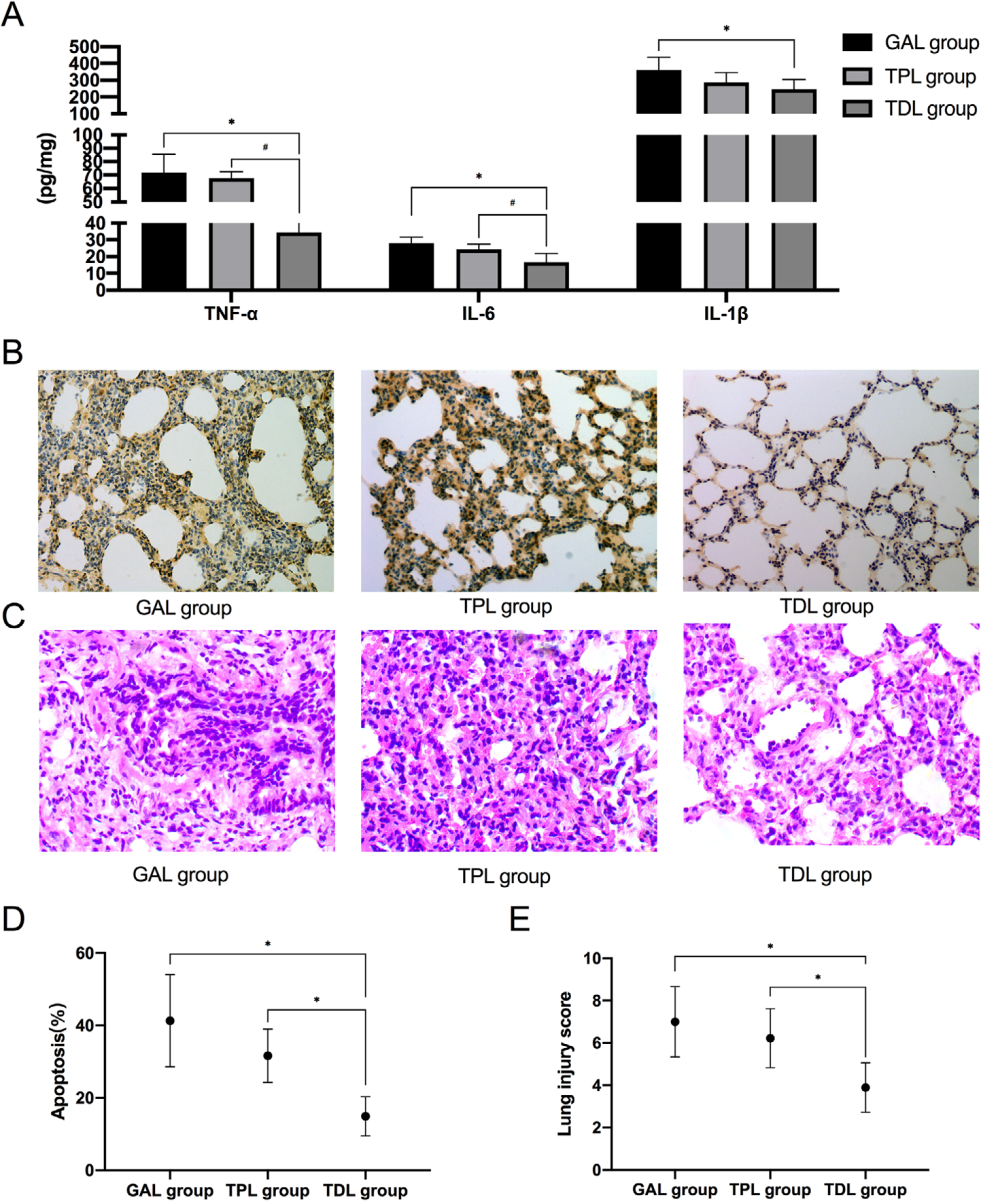
Recovery after surgery and anesthetic consumption results in lung cancer patients

#### 3.2.2 Postoperative recovery after lung surgery

Compared with that of the preoperative 6MWT, the 6MWT distance in the three groups was decreased at POD‐1, POD‐2, and POD‐3. The distance of the 6MWT at POD‐1 and POD‐2 in the TPE and TDE groups was greater than that in the GAE group (Figure 2B). No differences of EQ scores at postoperative day 30 were found among the three groups (Figure 2C). No differences in the incidence of chronic pain were found among the three groups (Figure 2D).

#### 3.2.3 Anesthetic consumption in lung surgery

Compared with that in the GAL group, the consumption of propofol and remifentanil was significantly decreased in the TPL group and TDL group. No differences were found between the TPL group and the TDL group.

#### 3.2.4 Inflammatory response

**FIGURE 3:**
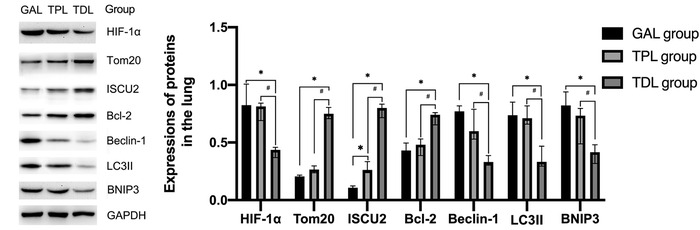
Representative images of lung injury, including injury scores, apoptosis, and inflammation

Compared with those in the GAL group and the TPL group, the concentrations of TNF‐α and IL‐6 were decreased in the TDL group. The concentration of IL‐1β in the TDL group was lower than that in the GAL group (Figure 3A).

#### 3.2.5 Apoptosis and injury in the lung surgery

The lung apoptosis index and injury scores were decreased in the TPL and TDL groups compared with the GAL group. The apoptosis index and injury scores in the TDL group were lower than those in the TPL group (Figure 3B‐E).

#### 3.2.6 Western blot in the lung tissue

**FIGURE 4:**
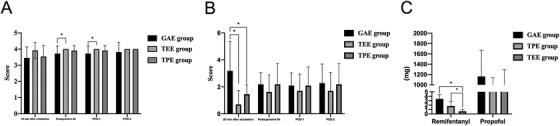
Representative images of protein expression by Western blot in lung cancer patients

The HIF‐1α, Beclin‐1, LC3II, and BNIP3 in the TDL group were lower than those in the GAL and TPL groups. Compared with those in the GAL group and the TPL group, the expression levels of Tom20, ISCU2, and Bcl‐2 were increased in the TDL group. The expression of ISCU2 was higher in the TPL group than in the GAL group (Figure 4).

### 3.3 Esophageal cancer patients

#### 3.3.1 Pain relief and sedation in the esophageal surgery

**FIGURE 5:**
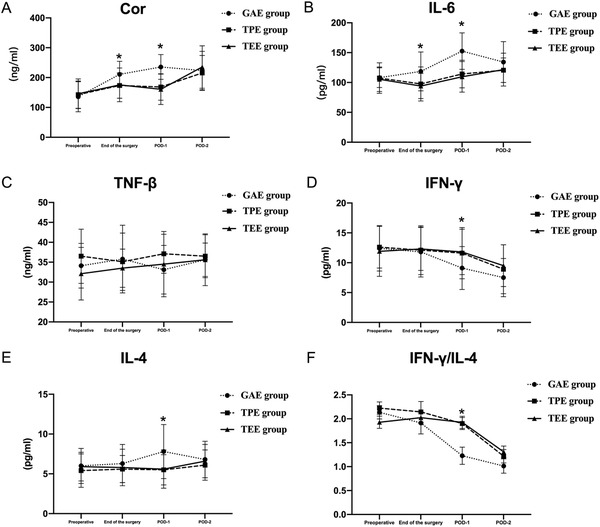
Pain relief, sedation and anesthetic consumption results in esophageal cancer patients

The SAS score was increased at postoperative 6 h and POD‐1 in the TEE group compared with the GAE group (Figure 5A). The postoperative acute pain scores at 20 min after extubation were decreased in the TPE and TEE groups compared with the GAE group (Figure 5B).

#### 3.3.2 Anesthetic consumption in the esophageal surgery

The remifentanil consumption was decreased in the TPE and TEE groups compared with the GAE group (Figure 5C). The TEE group consumed less remifentanil than the TPE group.

#### 3.3.3 Cytokine concentrations in the esophageal surgery

**FIGURE 6:**
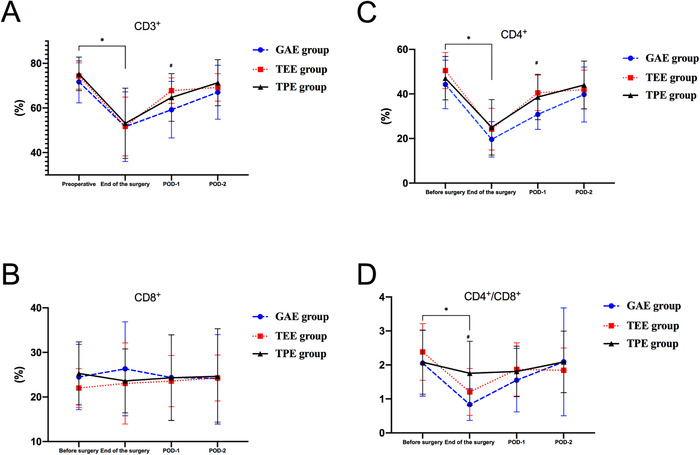
Cytokine concentrations by ELISA in esophageal cancer patients

Compared with those in the GAE group, the concentrations of Cor and IL‐6 were decreased at the end of the surgery and POD‐1 in the TPE and TEE groups (Figure 6A,B). Compared with that in the GAE group, the concentration of IL‐4 at POD‐1 in the TPE and TEE groups was decreased (Figure 6E). Compared with that in the GAE group, the concentration of IFN‐γ at POD‐1 and POD‐2 was increased in the TPE and TEE groups (Figure 6D). The ratio of IFN‐γ/IL‐4 at POD‐1 was increased in the TPE and TEE groups compared with the GAE group (Figure 6F).

#### 3.3.4 Lymphocyte subsets in the esophageal surgery

**FIGURE 7:**
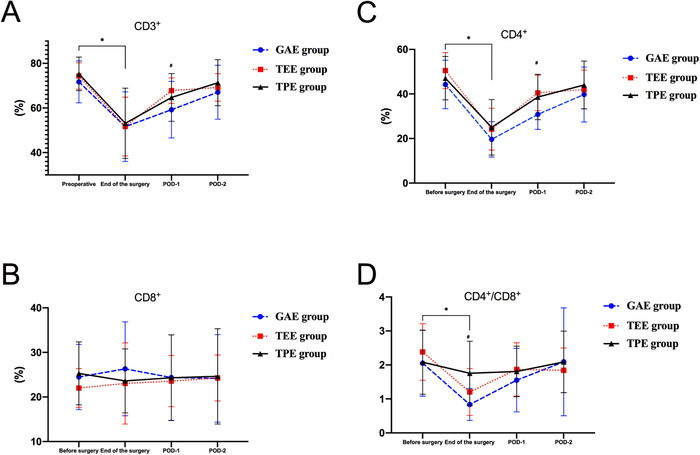
The percentage of lymphocytes in esophageal cancer patients

Compared with those at the preoperative time point, the percentages of CD3^+^ lymphocytes and CD4^+^ lymphocytes were decreased and then increased at POD‐1 and POD‐2 (Figure 7A,C). Compared with those in the GAE group, the percentages of CD3^+^ lymphocytes and CD4^+^ lymphocytes were increased in the TPE and TEE groups. No significant change in the percentage of CD8^+^ lymphocytes was found. The ratio of CD4^+^/CD8^+^ cells at the end of surgery was higher in the TPE group than in the GAE group (Figure 7D).

#### 3.3.5 Hemodynamics changes and early recovery in PACU

**FIGURE 8:**
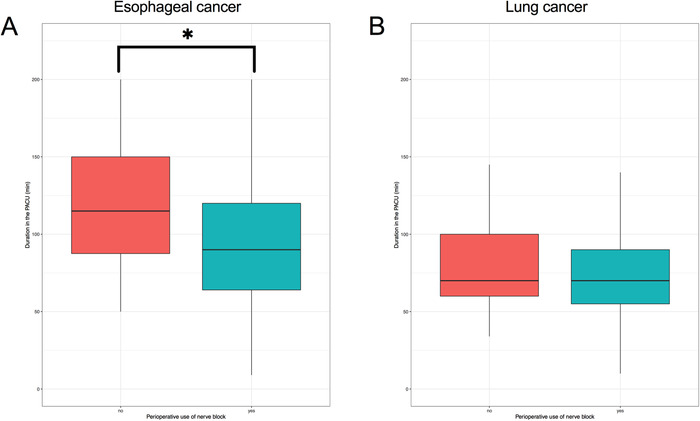
Perioperative hemodynamics changes

Compared with GAL group, the MAP was decreased in TPL group and TDL group at T_1_ (Figure 8A); the HR was decreased in TPL group and TDL group at T_1_, while higher HR was found in GAL group and TPL group than those in TDL group at T_2_ (Figure 8B). Compared with GAE group and TPE group, the MAP in TEE group was decreased at T_1_ (Figure 8C). Compared with GAE group, the HR was decreased in TPE group and TEE group was decreased at T_1_, while lower HR was found in TEE group than GAE group at T_2_ (Figure 8D). Significant differences were found among the three group in terms of vasoactive drugs in the lung cancer patients and esophageal cancer patients.

### 3.4 Retrospective results

**FIGURE 9:**
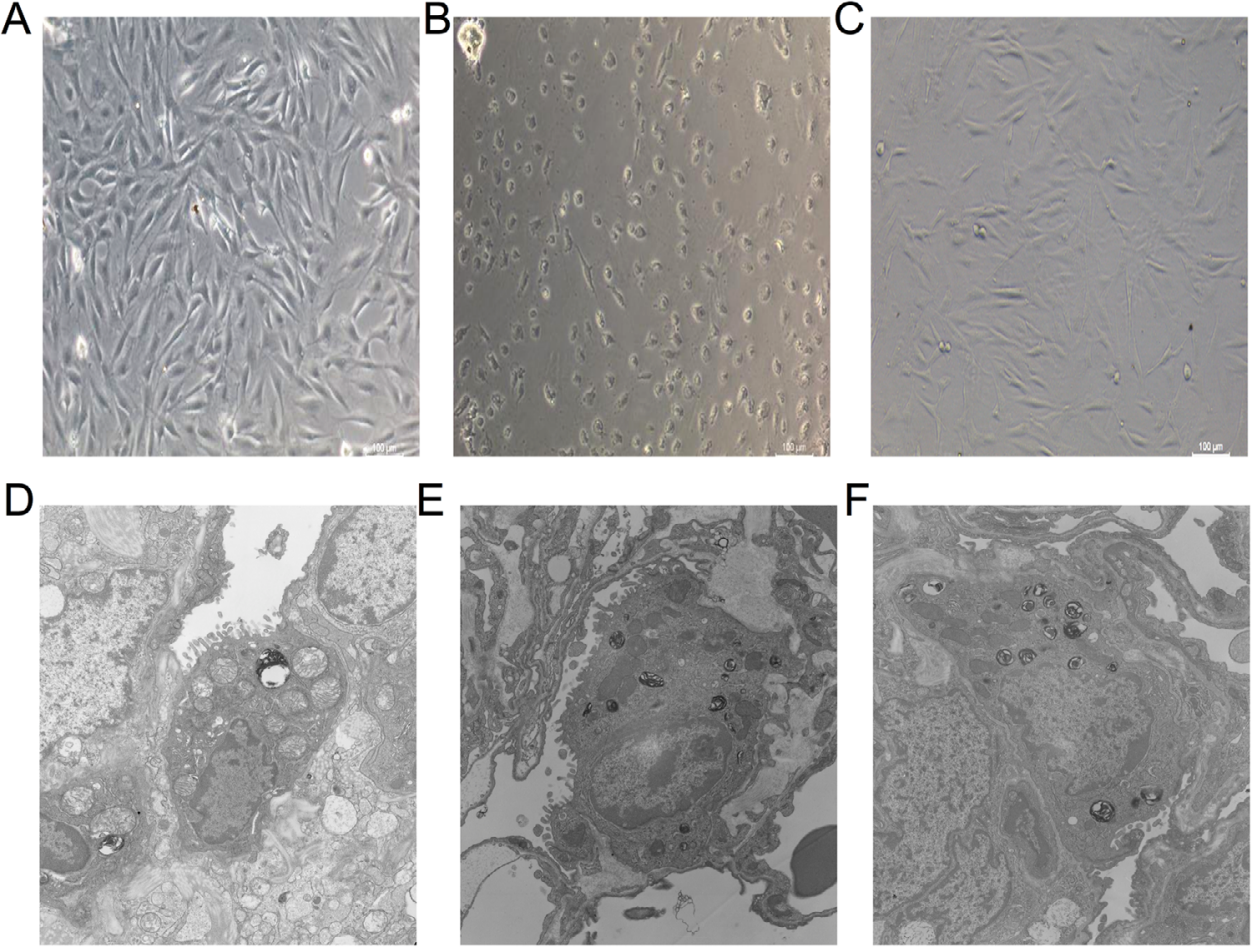
The Duration of patients in the PACU

A total of 814 patients with lung cancer and 411 patients with esophageal cancer were finally enrolled for analysis (Table 2). One‐way analysis of variance showed that TPVB significantly shortened the duration time in the PACU of patients with esophageal cancer (Figure 9A). Multivariate regression analysis showed that the application of TPVB in patients with esophageal cancer was a protective factor, shortening the duration in the PACU (estimate: −1.269, OR: 0.281, 2.5% CI: 0.078; 97.5% CI: 0.788; *P* = .028), and age was a risk factor that prolonged the duration in the PACU. Multivariate regression analysis showed that age, non‐minimally invasive surgery, and ASA III in patients with lung cancer prolonged the duration in the PACU.

**TABLE 2:** Multiple retrospective analysis results of duration in PACU

		Estimate	OR	2.5% CI	97.5% CI	*P‐*value
Lung cancer	Age	0.043	1.044	1.031	1.058	<.001
	Non‐Minimally Invasive Surgery	1.78	5.933	1.445	40.196	.027
	ASA III	0.396	1.485	1.012	2.189	.044
Esophageal cancer	TPVB	−1.269	0.281	0.078	0.788	.028
	Age	0.059	1.06	1.033	1.09	<.001
	Male	−0.906	0.404	0.23	0.686	.001

### 3.5 In vitro cell experiment

**FIGURE 10:**
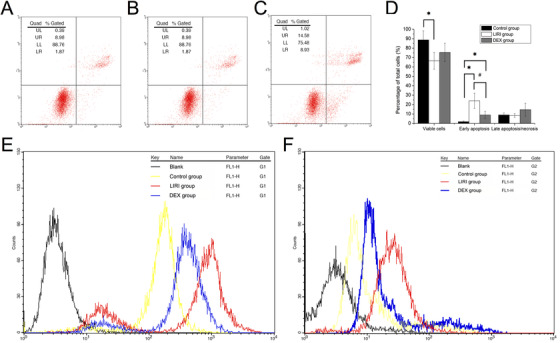
Mitochondrial morphological injury in the pulmonary epithelium was attenuated by DEX

Compared with the control group, the cell morphology of the LIRI group was retracted and rounded, and most of the cells died, whereas only a small proportion of the cells in the DEX group shrank and died (Figure 10A‐C). Mitochondrial injury was increased in the LIRI group but significantly reduced in the DEX group (Figure 10D‐F).

**FIGURE 11:**
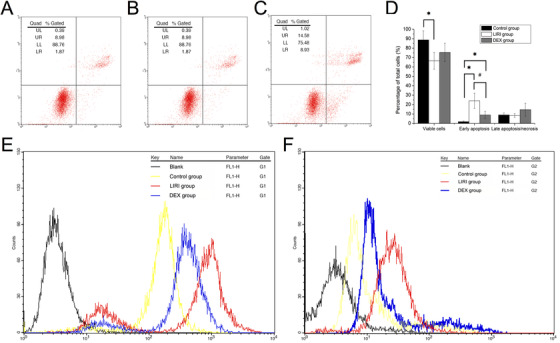
Mitochondrial functional injury in the pulmonary epithelium was attenuated by DEX

Apoptosis (Figure 11A‐C), ROS (Figure 10E), and mitochondrial membrane potential (Figure 11F) in the LIRI group and DEX group were higher than those in the control group. Compared with the LIRI group, the DEX group had more viable cells and decreased early apoptosis (Figure 11D). The ROS and membrane potential were lower in the DEX group than in the LIRI group (Figure 11E,F).

## 4 DISCUSSION

The main finding of this study was that thoracic paravertebral nerve block with or without dexmedetomidine provided much benefit to patients after thoracic surgery, including pain relief, enhanced recovery, improved pharmacoeconomics, improved immune function, and decreased lung injury and inflammation. The retrospective analysis results showed that, regardless of the analysis method, the PACU duration time of patients with esophageal cancer was significantly shortened by TPVB, and therefore, it is believed to be helpful in accelerating the recovery of patients. Mitochondrial injury induced by in vitro ischemia‐reperfusion was protected by DEX. To the best of our knowledge, this is the first study confirming that the lung injury and cellular immunity induced by surgery can be improved by TPVB combined with dexmedetomidine.

Thoracic nerve block could inhibit the pain and stress induced by the surgery, thereby a good stable hemodynamic could be expected. Our study has shown that MAP and HR could be decreased by TPVB, TPVB with paravertebral dexmedetomidine, and TEE. The application of vasoactive drugs was more commonly used in general anesthesia with no thoracic nerve block to patients with lung cancer and esophageal cancer. More vasoactive drug was used in epidural nerve block than paravertebral nerve block to patients with esophageal cancer. More stable hemodynamic could be achieved through TPVB or TPVB with paravertebral dexmedetomidine.

It is well recognized that thoracic paravertebral nerve block can alleviate the pain induced by thoracic surgery. In this study, TPVB reduced acute pain associated with pulmonary surgery and esophageal surgery, which is consistent with a previous study. Patients who received TPVB, TPVB combined with DEX, and TEA, especially pneumonectomy patients, gained enhanced pain relief. Better control of acute pain contributes to the incidence of chronic pain.^[^ ^10^ ^]^ A relatively high incidence of chronic pain was obvious after thoracic surgery.^[^ ^11^ ^]^ It has a profoundly negative impact on quality of life and produces psychological morbidities that include anxiety and depression. The incidence of chronic pain in this study was evaluated by an independent full‐time follow‐up staff. Multilevel single‐injection PVB may be a protective strategy for chronic post‐surgery pain at 6 months after breast cancer surgery.^[^ ^12^ ^]^ However, no differences in chronic pain were found in our study. The EuroQoL 5 Dimension 5 Questionnaire is a good predictor of quality of life.^[^ ^13^ ^]^ In accordance with the chronic pain level, the EQ scores were found to not be any different. These findings were not as encouraging as expected. The mechanism of chronic pain is complicated and includes many causative factors. Chronic pain could not be avoided by TPVB or DEX alone. The addition of DEX to local anesthetic at multiple levels of TPVB in patients undergoing VATS prolongs the duration of postoperative acute analgesia.^[^ ^14^, ^15^ ^]^ However, even TPVB combined with DEX in our study failed to decrease the development of chronic pain. A multimodal analgesia strategy might be needed to solve this problem.

It is widely recognized that the sooner bedside activities are undertaken, the better it will be for postoperative recovery. Pain is one of the most influential factors that hamper postoperative early bedside activity. Since postoperative pain could be inhibited by TPVB or TPVB with DEX, we assume that postoperative recovery could be thereby enhanced. The 6MWT is a simple and effective method for assessing patient cardiopulmonary function and is used to evaluate the quality of postoperative rehabilitation after pneumonectomy. The 6MWT is commonly used to measure exercise capacity and evaluate the postoperative rehabilitation.^[^ ^16^, ^17^ ^]^ In this study, the 6MWT was adopted to evaluate the rehabilitation ability. Compared with those at baseline, the walking distances at POD‐1, POD‐2, and POD‐3 were decreased in the three groups. TPVB or TPVB with DEX was proven to help improve the activity ability of patients. The bedside activity at POD‐1 and POD‐2 is of great importance to postoperative recovery. Based on this finding, TPVB or TPVB with DEX are recommended.

Perioperative pain and stress response can be alleviated by TPVB, TPVB with DEX, or TEA, thereby reducing anesthetic use. In this study, decreased consumption of propofol and remifentanil occurred in response to TPVB or TPVB with DEX in patients undergoing pulmonary surgery. In patients undergoing elective esophageal surgery, the consumption of remifentanil was also reduced by TPVB or TEA. Thus, less anesthetic is needed, and the pharmacoeconomics are very impressive.

### 4.1 Lung injury and inflammation

In this study, lung injury was assessed from the following aspects: local inflammation, injury score, apoptosis, and the measurement of certain related proteins. Systematic inflammation could be inhibited by the appropriate nerve block, including TPVB or TEA, or certain drugs, including DEX. A reduced systematic inflammatory response was achieved by the above strategy. Severe inflammation in the airway lavage fluid is harmful to the lung and associated with severe postoperative pulmonary complications. In our study, local cytokine levels in the lung tissue were examined. TNF‐α, IL‐6, and IL‐1β levels in the lung tissue were significantly decreased by TPVB with DEX. However, no such effect was found in the TPL group. TPVB could not inhibit local inflammation.

With prolonged single lung ventilation, independent lung injury, including apoptosis, is increased. Similar to the results of the inflammatory cytokines, TPVB with DEX significantly reduced the apoptosis and lung injury scores. TPVB did not alleviate apoptosis and lung injury. DEX has been shown to inhibit lung apoptosis and lung injury in previous studies. By examining the local lung tissue, we obtained similar results, which were consistent with those of previous studies.

To further verify these results, blood samples from patients who underwent esophageal cancer surgery were obtained to examine the levels of certain inflammatory cytokines.^[^ ^18^ ^]^ The concentrations of Cor and IL‐6 were reduced by both TPVB and TEA. TPVB and TEA produced similar inflammation inhibition effects. TPVB has been proven to be equal to TEA in many aspects, including pain relief and inflammation. Similar results were obtained in our study.

### 4.2 Potential mechanism

To further explore the potential mechanism of lung injury, certain proteins were examined. Previous studies have shown that hypoxia, apoptosis, and autophagy are involved in independent lung injury when one‐lung ventilation (OLV) is used. Therefore, several proteins associated with hypoxia, apoptosis, and autophagy were examined. Due to ethical requirements, lung tissue could be obtained only from the surgical specimens. HIF‐1α is a well‐known transcription factor that responds to hypoxia. The expression of HIF‐1α was inhibited by TPVB with DEX. Tom20 and ISCU2 are markers reflecting mitochondrial injury. The independent lung experiences relatively hypoxic conditions. Mitochondria play an important role in energy metabolism. Severe mitochondrial injury is detrimental to lung injury. TPVB with DEX improved the local hypoxia and protected the independent lung against mitochondrial injury.

Apoptosis and autophagy are involved in lung injury in the OLV model. Increased apoptosis and autophagy are observed in injured lungs. Apoptosis and autophagy inhibition are helpful in reducing lung injury. Bcl‐2 and Beclin‐1 are both regulators of autophagy and apoptosis.^[^ ^19^, ^20^ ^]^ Our results showed that TPVB with DEX contributed to the upregulation of Bcl‐2 expression and downregulation of Beclin‐1 expression. LC3II and BNIP3 are markers of autophagy and their expression was decreased by TPVB with DEX. Our study confirms that TPVB combined with DEX reduces apoptosis and autophagy.

### 4.3 Cellular immunity

Cellular immune function is decreased after esophagectomy.^[^ ^21^ ^]^ Decreased cellular immunity, which is regularly downregulated, plays a very important role in recovery after surgery, especially after tumor surgery. Maintaining reasonable perioperative immune function has become a key target for anesthesiologists. The levels of several cytokines and flow cytometry were used to evaluate cellular immune function.

The percentages of CD3^+^ and CD4^+^ T cells decreased after surgery and recovered gradually. Cellular immunity was proven to transiently decline in this study. Even at POD‐2, the T cell percentages did not recover to the baseline level. Interestingly, at POD‐1, the percentages of CD3^+^ and CD4^+^ T cells were higher in the TPE and TEE groups than in the GAE group. Our findings show that TPVB or TEA could contribute to the recovery of cellular immunity. The ratio of CD4^+^ and CD8^+^ T cells is regarded as a good indicator of cellular immunity. TPVB shows a significant advantage in maintaining immune function. TEA has no such advantage when compared with the GAE. The mechanism may lie in the relatively higher incidence of unstable hemodynamics associated with TEA.

Notable differences in IFN‐γ and IL‐4 between the groups occurred at POD‐1 and POD‐2. Immune function during the early postoperative period was clearly reduced, as has been proven by many studies. TPVB or TEA protected the immune function against a large decline in our study. This finding is in accordance with the results of the expression of T lymphocyte markers by flow cytometry. This finding again confirms that TPVB contributes to cellular immune functioning.

An additional retrospective analysis was conducted to determine the effect of TPVB on the recovery after thoracic surgery. The duration time in the PACU is a good indicator of early recovery from anesthesia and operation. The duration time in the PACU in patients with esophageal cancer was shortened by TPVB; thus, TPVB is a protective factor that could facilitate recovery in the PACU.

To further verify some results from the clinical research, an additional in vitro cell experiment was adopted. Mitochondrial injury after the lung ischemia‐reperfusion (LIRI) was mainly assessed. The pulmonary epithelium was severely damaged by LIRI. After treatment with DEX, the pulmonary epithelium injury was partially rescued, manifesting as changes in cell morphology, apoptosis, ROS, and mitochondrial membrane potential. Mitochondrial injury was reduced by DEX. This finding is consistent with the results from our clinical experiment.

In this study, several limitations should be noted. First, cellular immune function was not observed in lung cancer patients, and lung injury was not examined during esophageal cancer surgery due to the limited number of samples. The subgroup was not designed to be the same between the lung surgery and esophageal surgery groups. Whether or not corresponding conclusions can be drawn needs further research. Second, the follow‐up observation of clinical rehabilitation was not long enough. For patients, a 3‐ to 5‐year follow‐up time after cancer surgery is considered necessary. Third, the lung injury, inflammation, and cellular immunity protection mechanisms need further investigation. Preliminary results and potential mechanisms are discussed in this study. More in vitro and animal studies should be carried out to explore the specific mechanisms.

Overall, three commonly used nerve block methods (TPVB, TPVB combined with DEX, and TEA) were used in this study. Clinical rehabilitation, lung injury, local and systematic inflammation, and cellular immune functions were the four main objectives investigated in this study. TPVB, TPVB combined with DEX, and TEA were confirmed to enhance the recovery after surgery. More stable hemodynamic could be achieved by TPVB than TEE. TPVB was comparable to TEA in reducing acute pain and inflammation in esophageal surgery. The cellular immune function was better preserved by TPVB than by TEA. In lung surgery, TPVB combined with DEX was comparable to TPVB on for acute pain relief, early bedside activity, and the incidence of chronic pain. TPVB combined with DEX during lung surgery showed superiority to TPVB in alleviating lung injury, inflammation, and apoptosis.

## DECLARATIONS

Ethics approval and consent to participate: This prospective study was approved by the Ethics Committee of Henan Provincial People's Hospital, and written informed consent was obtained from all patients.

## AVAILABILITY OF DATA AND MATERIALS

The datasets used and analyzed during the current study are available from the corresponding author on reasonable request.

## CONFLICT OF INTEREST

The authors declare no conflict of interest.

## FUNDING INFORMATION

Provincial‐Ministry Co‐construction Project of Medical Science and Technology Project of Henan Province (SB201901091); Henan provincial science and technology research project (2018020844); The funding body played the roles in the design of the study and collection, analysis, and interpretation of data and in writing the manuscript.

## AUTHOR CONTRIBUTIONS

W.Z. wrote the manuscript and analyzed the data. X.C. were associated with sample detection and measurements. L.Z., B.L., and H.G. collected the human samples and were associated with data acquisition. M.S. was associated with sample detection and measurements. J.G. guided and supported the project. J.Z. was associated with design and implementation. All authors have read and approved the final version of the manuscript.
